# Designing the First Pregnancy Guaranteed Income Program in the United States: Qualitative Needs Assessment and Human-Centered Design to Develop the Abundant Birth Project

**DOI:** 10.2196/60829

**Published:** 2025-01-27

**Authors:** Deborah Karasek, Jazzmin C Williams, Michaela A Taylor, Monica M De La Cruz, Stephanie Arteaga, Sabra Bell, Esperanza Castillo, Maile A Chand, Anjeanette Coats, Erin M Hubbard, Latriece Love-Goodlett, Breezy Powell, Solaire Spellen, Zea Malawa, Anu Manchikanti Gomez

**Affiliations:** 1 School of Public Health Oregon Health & Science University and Portland State University Portland, OR United States; 2 Department of Obstetrics, Gynecology, and Reproductive Sciences University of California, San Francisco San Francisco United States; 3 University of California, San Francisco San Francisco, CA United States; 4 Expecting Justice San Francisco, CA United States; 5 Sexual Health and Reproductive Equity Program School of Social Welfare University of California, Berkeley Berkeley, CA United States; 6 Department of Human Ecology Univeristy of California, Davis Davis, CA United States; 7 California Preterm Birth Initiative University of California, San Francisco San Francisco, CA United States

**Keywords:** maternal and child health, economics, public health, qualitative research methods, programs (evaluation and funding), community-centered, pregnancy, first pregnancy, behavioral interventions, racial health, financial stress, Abundant Birth Project, infant health, infant, Black

## Abstract

**Background:**

Racial inequities in pregnancy outcomes persist despite investments in clinical, educational, and behavioral interventions, indicating that a new approach is needed to address the root causes of health disparities. Guaranteed income during pregnancy has the potential to narrow racial health inequities for birthing people and infants by alleviating financial stress.

**Objective:**

We describe community-driven formative research to design the first pregnancy-guaranteed income program in the United States—the Abundant Birth Project (ABP). Informed by birth equity and social determinants of health perspectives, ABP targets upstream structural factors to improve racial disparities in maternal and infant health.

**Methods:**

The research team included community researchers, community members with lived experience as Black or Pacific Islander pregnant, and parenting people in the San Francisco Bay Area. The team conducted needs assessment interviews and facilitated focus groups with participants using human-centered design methods. Needs assessment participants later served as co-designers of the ABP program and research, sharing their experiences with financial hardships and government benefits programs and providing recommendations on key program elements, including fund disbursement, eligibility, and amount.

**Results:**

Housing affordability and the high cost of living in San Francisco emerged as significant sources of stress in pregnancy. Participants reported prohibitively low income eligibility thresholds and burdensome enrollment processes as challenges or barriers to existing social services. These insights guided the design of prototypes of ABP’s program components, which were used in a design sprint to determine the final components. Based on this design process, the ABP program offered US $1000/month for 12 months to pregnant Black and Pacific Islander people, selected through a lottery called an abundance drawing.

**Conclusions:**

The formative design process maximized community input and shared decision-making to co-design a guaranteed income program for Black and Pacific Islander women and people. Our upstream approach and community research model can inform the development of public health and social service programs.

## Introduction

### Background

Pregnancy is a critical period of development when stress can have deleterious effects on both the pregnant person and their baby, leading to adverse birth and long-term health outcomes [1,2] that disproportionately affect Black, Indigenous, and Native Hawaiian or Pacific Islander people [3,4]. In the United States, Black infants are twice as likely to be born preterm or with low birth weight compared to White infants [3]. These inequities extend to maternal health, with Black women being 3.3 times more likely to die during or after pregnancy than White women [5]. While rates of adverse outcomes are often obscured by aggregation of data among Asian and Pacific Islander subgroups [6], Native Hawaiian and other Pacific Islander people experience higher rates of low birth weight, preterm birth, and macrosomia [3,7,8]. Even in San Francisco, a wealthy, well-resourced city, the shrinking Black and Pacific Islander populations experience significant inequities in birth outcomes [9]. Black and Pacific Islander pregnant women and people experienced the highest rates of preterm birth, at 12.7% and 8.9%, respectively, in 2014-2016, the last years with enough births to Pacific Islander people to reliably report [10]. Research increasingly indicates that racism—structural, institutional, and interpersonal—is a key underlying driver of these inequities [11,12]. It is unsurprising, therefore, that clinical, educational, and behavioral interventions have failed to meaningfully narrow racialized birth disparities [3,13-15].

Efforts to reduce inequities must address exposure to interpersonal, institutional, and structural racism as the root cause of disproportionate risk [16-19]. Racial inequity in income and wealth is one manifestation of structural racism, with important public health ramifications [16,20]. The racial wealth gap has resulted from a long history of exclusion of Black families from economic opportunity, through a combination of redlining, exclusionary zoning, subprime loans, and “urban renewal” programs, among other tactics [21]. This leaves Black families and other families of color more vulnerable to economic stress during pregnancy. Financial stress may be particularly acute during pregnancy, a period of income volatility when families frequently experience changes in housing, employment, health care, and childcare costs [22]. Indeed, in a nationally representative study of pregnant and postpartum women, over half reported general financial stress [23]. Yet, efforts to directly alleviate poverty and financial stress to address birth inequities are limited. In San Francisco, the median annual income in 2020 for White households was US $146,043, while it was just US $38,862 for Black households and US $90,917 for Pacific Islander households [24], making it the city with the third largest income disparity in the United States [25]. The Insight Center Family Needs calculator estimated the minimum income required to live in San Francisco in 2021 without public or private assistance as US $60,232 for a single adult, US $120,519 for a single adult with 1 preschooler, and US $167,432 for a household with 2 adults, 1 infant, and 1 preschooler [26]. To meet the needs of a family of three, Black and Pacific Islander households would have needed to earn 4 and 2 times their current average incomes, respectively.

Guaranteed income describes the disbursement of unconditional cash to individuals or families in a community without restrictions, allowing flexibility to close gaps where needs are not being met by public benefits programs [27]. Guaranteed income programs offer the potential to address poverty and stress caused by economic racism and the racial wealth gap. Philanthropic funders, cities, and states, including California, are increasingly investing in guaranteed income as a poverty reduction and equity-generating strategy [28]. Emerging findings from the growing research on United States guaranteed income pilots have found that monthly income supplements improve the sense of trust in institutions, dignity, and autonomy and increase savings, without changing spending patterns [29,30]. Magnolia Mothers Trust, a program providing US $1000 per month for 12 months to Black mothers living in federally subsidized housing in Jackson, Mississippi, allowed mothers to save for emergencies, buy food, pay childcare expenses, and prioritize their health [30].

At the time of the Abundant Birth Project’s (ABP) inception, there were no guaranteed income programs in the United States that addressed the critical period of pregnancy. However, evidence from other contexts supported a guaranteed income in pregnancy as a health intervention. A quasi-experimental study among 14,591 pregnant women in Manitoba, Canada, found that an approximately 10% increase in income from a pregnancy income benefit resulted in reductions of low birthweight by 21% and preterm birth by 18% [31,32]. Since the launch of ABP, there has been a proliferation of guaranteed income programs across California, including other programs that target pregnancy [28,33,34].

### The Abundant Birth Project

ABP is a program of Expecting Justice, a Black-led, cross-sector collaborative focused on birth equity in San Francisco that includes mothers, public health professionals, health and social systems partners, and governmental leaders. Expecting Justice uses a collective impact model, which is a process to bring together diverse stakeholders to create a common agenda, establish shared measurement, and foster mutually reinforcing activities for stakeholders [35]. As part of its work to advance birth equity, Expecting Justice is guided by community voice and emphasizes upstream solutions that address the social determinants of health. As defined by the National Birth Equity Collaborative, birth equity is the assurance of optimal birth conditions for all people, with an ongoing effort to address racial and social inequities [36]. The social determinants of health result from the larger systems at play, such as economic policies, racism, and political systems that shape the conditions of daily life and health [37,38]. These frameworks informed the focus on this pregnancy-guaranteed income program as an equity-generating strategy to directly address income insecurity as a social determinant of health by interrupting the pathway from economic exclusion and stress to adverse birth outcomes. Expecting Justice targeted this intervention toward communities most likely to experience preterm birth to help narrow disparities in preterm birth rates. Focusing on the most marginalized aligns with the birth equity framework, emphasizing that targeted support, rather than universal availability, is essential for addressing birth inequities [36].

Expecting Justice entered a community-academic partnership with the University of California, Berkeley, and the University of California, San Francisco, to design and evaluate ABP. We developed the ABP program and evaluation by actively, meaningfully, and intentionally centering the voices and experiences of the communities most affected by the adverse birth outcomes the program seeks to improve. ABP launched in June 2021, with the goal of enrolling 150 Black and Pacific Islander pregnant women and people (including gender-diverse individuals with the capacity for pregnancy) over 18 months.

This paper describes the formative research process, under the co-leadership of community members, used to design ABP.. Our primary objectives were to (1) determine the financial needs of Black and Pacific Islander women and people during pregnancy, and (2) collaboratively design a pregnancy-guaranteed income pilot program with Black and Pacific Islander mothers. We hope that the process and results from the formative research to design ABP will be useful to other researchers, practitioners, and community groups developing interventions to address racial health disparities, including guaranteed income pilot programs.

## Methods

### Community Research Model

ABP used shared decision-making and community-centered practices in all phases of the program development and evaluation, by purposely centering the voices of community members with lived expertise [39]. As a community-based participatory research project, ABP recognized that this relationship is dynamic and requires continual effort to rebalance traditional power structures [40]. To support these efforts, 4 community researchers (CRs) served as key members of the community-academic partnership. The CRs are Black and Pacific Islander community members who have lived experience of pregnancy and parenting in the San Francisco Bay Area. They come from a variety of professional backgrounds and have an interest in learning or experience using research methods to advance birth equity. We adopted a shared power structure and decision-making process that included following Expecting Justice’s “expert opinion protocol” for all final decisions. This protocol enabled ABP stakeholders who identified as Black and/or Pacific Islander mothers to have protected time to share their insights with the larger group without contradiction before voting. We also used weighted voting in decision-making to prioritize the perspectives of those who identified as Black and/or Pacific Islander mothers.

**Figure 1:**
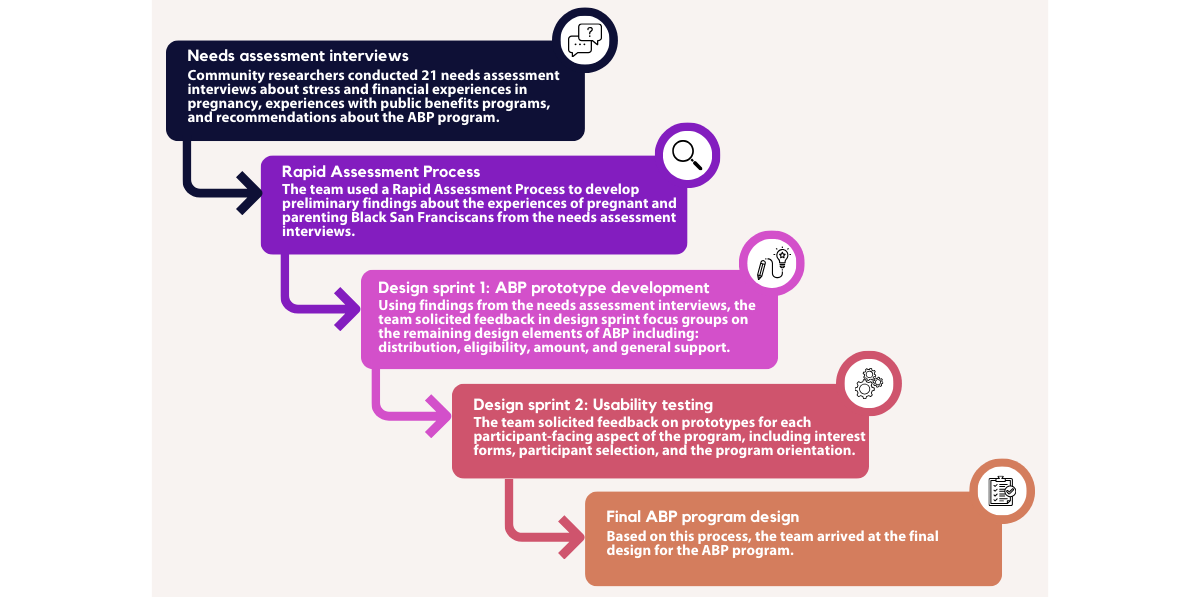
The design process for the Abundant Birth Project involved conducting needs assessment interviews, an RAP to analyze findings, a prototype development design sprint, and a usability design sprint to arrive at the final design. ABP: Abundant Birth Project.

The process of designing the ABP program and evaluation involved (1) conducting interviews as part of the needs assessment, (2) a Rapid Assessment Process (RAP) for analysis, (3) a prototype development design sprint, and (4) a usability testing design sprint (Figure 1). The CRs contributed to the full process, bringing expertise from their lived experience with pregnancy and parenting in the Black and Pacific Islander communities: developing research questions, formulating survey and interview instruments, recruiting participants, conducting interviews, facilitating design sprints, interpreting results, and making key program decisions. CRs received training in data collection and analysis supported by the university-based staff, which coupled with the team’s shared power structure, helped to dismantle the hierarchy often present in research institutions that keeps community members out of decision-making processes [41,42]. Expecting Justice also led an ABP working group with birth equity stakeholders, including Black and Pacific Islander mothers, community organization representatives, social service providers, maternal health leaders and researchers, and representatives from health plans to collaborate and provide strategic resources to support program development. The ABP working group met monthly to inform key program design and implementation decisions before, during, and following the formative phase.

The shared decision-making structure with the ABP program and research teams, CRs, and ABP working group led to tangible improvements in research and program design. For example, the CRs voiced concerns that research studies rarely compensate participants in a way that truly values their time. Under their leadership, the team increased the amount of participant remuneration for the interviews and design sprints from US $50 to US $75 and US $100 to US $200, respectively.

### Needs Assessment Interviews

To inform ABP’s design, we first conducted interviews to understand the experiences Black and Pacific Islander women and people face during pregnancy in San Francisco. Inclusion criteria were aged 18 years or older, identified as Black and/or Pacific Islander, spoke English, and either currently pregnant or had been pregnant while residing in San Francisco in the past 5 years. Participants were recruited from social media and local community organizations. The CRs closely collaborated on the development of the interview guide to ensure that language and questions would capture appropriate topics in a comfortable environment for participants. We conducted interviews from April to June 2020. The CRs conducted the interviews using a web-based videoconference platform or by telephone. Interviews were recorded and professionally transcribed.

The interview guide addressed stress and financial experiences during pregnancy and experiences with and perception of public benefits programs. We also asked participants for recommendations on the ABP program design, including how much money would alleviate financial stress during pregnancy, how to best distribute the income supplement, and program eligibility criteria and restrictions.

Before conducting interviews, the CRs underwent training in qualitative research methods led by Dr Brittany Chambers, a Black scholar, mother, and community engagement expert. The training included an introduction to asking research questions, qualitative data collection, and interviewing skills. The entire research team went through the training together, building trust and rapport throughout the process. The CRs described the importance of being trained by a researcher from their own communities. Dr Chambers used art-based activities that allowed the CRs to celebrate their stories and the whole team to tap into their imagination and creativity and engage with one another outside of hierarchies. CRs received compensation for their time and expertise in the training.

### About RAP

To identify key topics, we used RAP, an intensive, team-based, iterative analytic approach that uses data triangulation to develop preliminary findings from the perspective of an insider—pregnant and parenting Black and Pacific Islander San Franciscans [43]. RAP is well-suited for qualitative research informing the design of interventions, as it allows for rapid and timely data reduction and ongoing analysis during data collection. We brought together the team, including CRs, program staff, and student research assistants for shared qualitative analysis training. CRs, other research team members, and program staff used summary templates to create a memo for each interview. Members of the research team reviewed memos for consistency. The first step of data reduction involved matching domains from the summary template to interview questions. The domains included concerns about stress and pregnancy, employment, experiences with social services, desired programs and services, feelings about the income supplement, recommended supplement amount, eligibility restrictions, disbursement, recruitment recommendations, and additional financial services. Finally, we used an individual-level data matrix to synthesize key data across participants and identify themes.

### Human-Centered Design Sprints

#### Overview

Using design thinking methodology, we conducted 2 focus groups, called human-centered design sprints, to develop and refine the facets of the guaranteed income program following themes identified from the need assessment interviews. Originating in consumer technology, design thinking is a method of developing innovative solutions and products with leadership and input from the end user [44]. A design sprint refers to a rapid, human-centered design activity, in which individuals are brought together to discuss and iterate on program design components [45]. Design sprints prioritize qualitative feedback from stakeholders and target end users, and encourage creative thinking [45]. Human-centered design methodology is especially well-suited for developing urgently needed interventions to reduce health inequities and has previously been used to gather community input on solutions for perinatal disparities [46]. We partnered with design thinking firms that helped determine the structure of the design sprints and trained the ABP program staff and CRs on how to facilitate these sessions.

Due to the COVID-19 pandemic, we conducted the design sprints using videoconferencing software and recorded the sessions. We provided tablets with internet connectivity to all design sprint participants who lacked internet access to participate virtually. For continuity, we invited participants from the needs assessment interviews to participate in the design sprints. Co-designing with the same participants also ensured that their voices and perspectives were reflected in the program prototypes. Each design sprint session was 4 hours.

#### Design Sprint 1: ABP Prototype Development

Guided by findings from the needs assessment interviews, our team determined 4 remaining design questions to be answered through the first design sprint.

1. Distribution: How can we best deliver money in a way that is both inclusive and convenient?
2. Eligibility: How can we best serve the Black and Pacific Islander communities in San Francisco while allowing for flexibility in eligibility criteria?
3. Amount: How can we balance financially supporting pregnancy needs with the desire to support as many people as possible?
4. General support: How can we support pregnant people, not just financially, but holistically, during their pregnancy?

**Figure 2:**
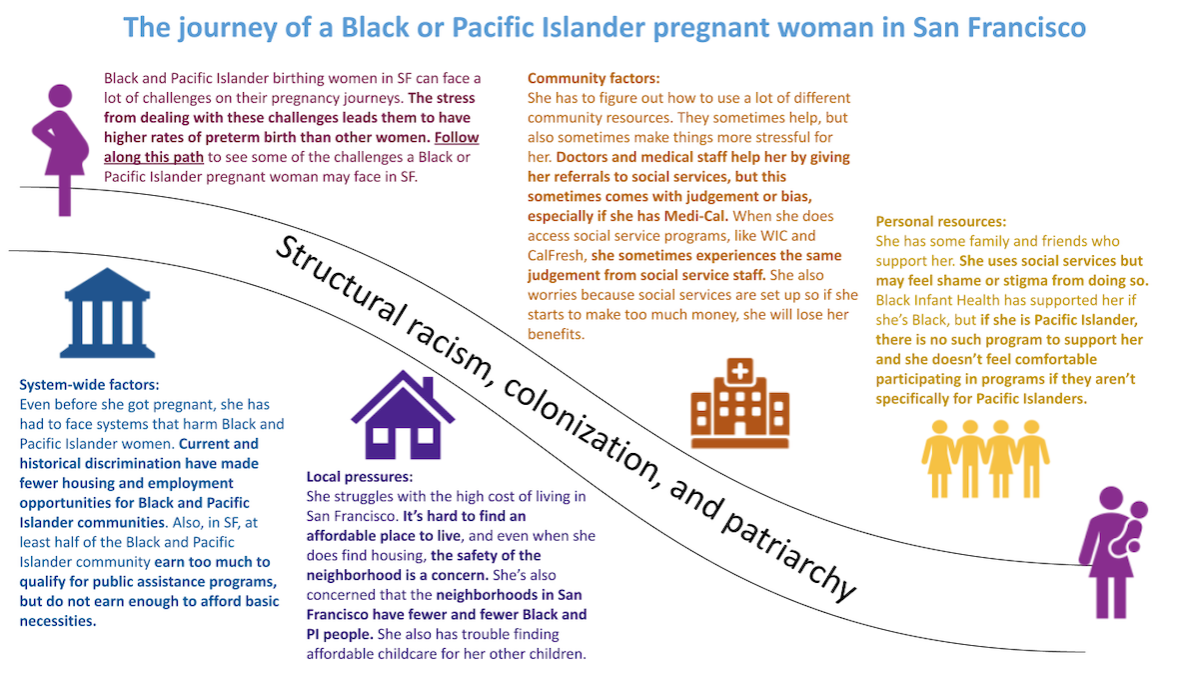
The journey map was developed using themes from needs assessment interviews conducted from April to June 2020 for the Abundant Birth Project. It described how racism contributes to financial and housing challenges, stress, and stigma for Black and Pacific Islander pregnant women in San Francisco. SF: San Francisco; PI: Pacific Islander; WIC: Women, Infant and Children.

Before the design sprint, we provided participants with a welcome packet to introduce ABP and the purpose of the session. The welcome packet included a journey map to contextualize the human-centered design sprints for participants (Figure 2). The journey map was developed using common themes from the needs assessment interviews to guide participants through a story of a Black or Pacific Islander pregnant woman in San Francisco, focusing on how racism contributes to financial and housing challenges, stress, and stigma.

Following the first design sprint, we synthesized feedback from participants with findings from the needs assessment interviews to develop ABP program prototypes. Specific recommendations from participants in interviews and the first design sprint informed the prototypes for key components of ABP, including distribution method, amount, and eligibility criteria. The themes surrounding previous experiences with social services informed components of the ABP prototype that focused on support that should be offered in addition to the supplement itself.

#### Design Sprint 2: Usability Testing

Using the learnings from the first design sprint, our team developed prototypes for each participant-facing aspect of the program, including the interest form, eligibility interview, participant selection, program enrollment, orientation, program experience, and research engagement. Design thinking is iterative; therefore, the second design sprint focused on usability testing with the ABP prototypes. Participants gave feedback on the prototypes, which helped ensure that the final program design aligned with community values and perspectives. CRs facilitated breakout groups that focused on each aspect of the program experience. For research engagement, participants were asked about what concerns they would have about engaging in research and what would make them excited to opt into the research. We made final decisions regarding the prototypes using Expecting Justice’s “expert opinion” protocol.

### Ethical Considerations

The institutional review board of the University of California, San Francisco, approved this study’s protocol (IRB #19-27608, reference #267614). Participants signed consent forms for interviews and design sprints after reviewing them with study personnel. The research team removed identifiers (eg, names) from interview transcripts to deidentify data. Participants received a US $75 gift card incentive for taking part in the needs assessment interviews and a US $200 gift card incentive for participation in each design sprint.

## Results

### Needs Assessment Interviews

#### Participant Demographics

We conducted 21 in-depth interviews from April to June 2020. Seventeen participants identified as Black or African American, 5 as Pacific Islander, 2 as Latinx, and 1 as Native American or Indigenous (Table 1). All 21 participants identified as women. Eleven participants had experienced homelessness in the past, and 15 were at risk of food insecurity [47].

**Table 1:** Selected demographics for Abundant Birth Project formative research participants in San Francisco, California. Formative research needs assessment interviews and design sprints occurred from April to December 2020 (N=21).

Characteristic		Value, n (%)
**Racial identity^a^**		
	Black or African American	17 (81)
	Latinx	2 (10)
	Native American or Indigenous	1 (5)
	Pacific Islander	5 (24)
**Pregnancy status**		
	Pregnant at the time of interview	6 (29)
	Pregnant in the last 5 years	15 (71)
**Income**		
	100% federal poverty level	7 (33)
	100%-200% federal poverty level	6 (29)
	>200% federal poverty level	6 (29)
	Do not know	2 (9)

^a^Racial identity categories are not mutually exclusive.

#### Experiences of Financial Stress in Pregnancy

All participants discussed feeling stressed during their pregnancies; sources included negotiating employment leave, the health of their baby and themselves, and most frequently, housing and the high cost of living in San Francisco. Some participants reported being housing insecure or homeless during their pregnancies, underscoring the precarity of meeting basic needs in San Francisco. One participant described how the cost of living in the Bay Area was a primary driver of her stress.

Especially living in San Francisco Bay Area, everyone is so uptight and on edge because it's so expensive to live here, and we're always stressed-out about working or money and just trying to make it, so with all of that already going on top of bringing another life into the world, things can get really, really stressful.

Currently pregnant, identified as Black and Latinx

Another participant discussed her distressing personal experience with homelessness and the tradeoffs she had to consider to keep her child safe.

I was couch surfing. I was homeless. I became homeless March of 2019, so I was couch surfing waiting for a shelter to come about or some type of services, so I’ve been just couch surfing, sleeping on the…well, I will sleep at the bus stop, but my son would be with one of my friends during the night, so that he would [be] able to sleep comfortably.

Last pregnant in 2020, identified as Pacific Islander

Participants discussed the need for assistance programs that could include middle-income families. One participant underscored this by saying:

The hard part is being in this random middle class just because of my husband's one job, even though nobody's taking into account all of our expenses and things. And so, it's still kind of like we're low-income, but it doesn't read low-income on paper.

Last pregnant in 2018, identified as Black, Latinx, and Native American or Indigenous

Other participants who considered themselves middle-income echoed this “catch-22” situation, in which they struggled financially because they did not make enough to meet the costs of living in the San Francisco Bay Area, but their income surpassed the federal poverty line used to calculate public benefit eligibility, rendering them ineligible for assistance. Describing this impossible situation, one participant said:

Just because someone has a job doesn’t mean that they shouldn’t not qualify and get this extra help, because they still have stress. They still are worried about something financially, you know…what I’m saying is like most programs if you make less than $35,000 a year. Well, that’s poor, poor, poor in San Francisco. But what about like the person who makes $32 an hour, you know, that can’t make ends meet or is stressing and struggling. Like there’s never any kind of help or support for them. They can’t get the Medi-Cal. They can’t get the WIC. No welfare. Children’s Council. None of that.

Most recently pregnant in 2019, identified as Black

#### Experiences With Government Benefits Programs

Most participants (18/21) had experiences with government benefits programs, including CalFresh (California’s Supplemental Nutrition Assistance Program), CalWorks (California’s Temporary Assistance to Needy Families program, commonly known as “welfare”), Medi-Cal (California’s Medicaid program), the Special Supplemental Nutrition Program from Women, Infant and Children, and Black Infant Health, a statewide program to improve the health of Black mothers and babies. Participants found most government benefits programs financially helpful and were grateful for the aid. One participant said:

CalFresh helped a lot. It was a lifesaver, actually. We were just so broke at that time and paying San Francisco rent and everything, so that definitely helped us get by until I got a more full-time job, and things changed after that, but it definitely got us through.

Currently pregnant, identified as Pacific Islander

Despite appreciating the financial assistance, participants found the application processes for many benefits programs burdensome and income eligibility requirements too restrictive, given the cost of living in San Francisco. Additionally, participants reported feeling stigmatized by the benefits program staff or when using certain resources, such as a CalFresh Electronic Benefit Transfer card at a grocery store or Medi-Cal at their health care provider. Describing previous stigmatizing experiences while using Electronic Benefit Transfer at the grocery store, one participant said:

I feel like you’re looked at differently, you’re really treated differently. Like, I would have people, like, cashiers, kind of put me to the side like, ‘Oh, figure out your check stuff, and then let me get these people that are paying.’ Like, I’ve had people say that to me, and so that part of it is just like—and then, you know, [the cashiers] getting up on a loudspeaker—just it just, all that stuff, nobody’s discreet about anything…

Most recently pregnant in 2016, identified as Black

#### Recommendations for a Pregnancy Guaranteed Income Program

Overall, participants enthusiastically supported the idea of an income supplement for Black and Pacific Islander people during pregnancy, feeling that the flexible resources would significantly reduce stress during pregnancy.

That would be so helpful and also show compassion towards the women [and] what they go through because people don't know really what we go through unless you had the baby...that money would definitely be helpful towards whether or not you pay for diapers or food, you know what I'm saying. Like sometimes you can't do both, but you need both, right?

Most recently pregnant in 2018, identified as Pacific Islander

We asked participants how much money per month they believed would reduce financial stress during pregnancy. Three participants felt “anything would help,” 4 recommended less than US $500, 11 recommended an amount between US $700 and US $1000, and 4 believed more than US $1000 would be necessary due to the high cost of living in San Francisco. Participants recommended electronic fund distribution for speed and because many bills are paid on the web. They felt that cash may be spent too quickly, makes tracking spending difficult, and cannot be replaced if lost. Some also expressed concerns that cash could not be used for remote or web-based purchases, which was essential during the data collection period at the beginning of the COVID-19 pandemic. Many mentioned the need for an option for individuals without a bank account. When asked about potential other services that should be offered alongside ABP, most felt it would be valuable to offer optional support with finances (eg, credit building or repair, savings, banking, and taxes), childcare, and housing.

Some participants expressed concerns about how recipients would spend the income supplement. This may reflect false and racist narratives about how Black mothers use public assistance programs [48]. Often speaking in generalities, participants discussed concerns that income supplement recipients may not use the money in ways society deems “right.” Participants also reported concerns that if hypothetical recipients misused the funds, it may result in the cessation of the program.

Of course, we have those folks who would abuse it and maybe not really using it, well, I guess if it's no strings attached, it doesn't matter what they use it for, but I guess I would just be afraid of anything happening like that so where it would be abused to a point that we'd lose it altogether, you know what I mean, so that would be a concern.

Most recently pregnant in 2018, identified as Pacific Islander

Several participants discussed eligibility requirements, such as an income cap or debt-to-income ratio. These criteria were proposed to ensure that the limited resources are directed to the target population.

### Design Sprint 1: ABP Prototype Development

#### Overview

Fifteen interviewees from the needs assessment participated in the first design sprint, which occurred in July 2020. The CRs facilitated breakout sessions to discuss the concept of an income supplement and address the 4 key program design questions.

#### Amount

Participants believed that a monthly supplement should be at least US $1000 to provide a noticeable benefit during pregnancy. As participants also wanted the program to accommodate as many people as possible, they preferred a supplement to reach 150 participants over a larger supplement reaching fewer people.

#### Disbursement

**Figure 3:**
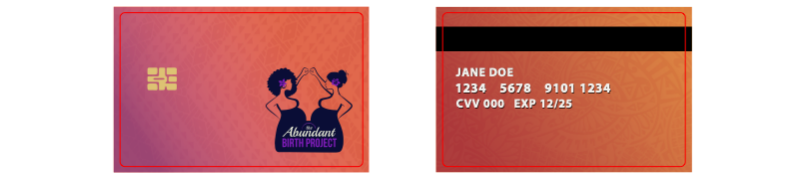
The Abundant Birth Project debit card included imagery that reflected participants’ desire for a beautiful design to prevent feelings of stigma or judgment often associated with using other benefits programs.

Some participants recommended multiple payment types for the disbursement of funds. As this was not feasible with the disbursement organizations, participants came to the consensus that an ABP-specific debit card (as opposed to cash, check, or direct deposit) would be the most acceptable way to distribute the supplement. Participants discussed what would make the debit card the most accessible, such as not requiring a bank account or social security number. They also expressed they wanted the ABP debit card to have a beautiful design that reflected their communities, so recipients would not feel stigmatized or judged as they often did in other benefits programs (Figure 3).

#### Eligibility

Participants felt the program should have additional eligibility criteria aside from identifying as Black and/or Pacific Islander, such as gestational age in pregnancy and income. For income, participants expressed a desire for the program to serve individuals who do not qualify for government benefits but still struggle to meet their needs in San Francisco. Design sprint participants believed that ABP participants should be randomly selected to give everyone interested an equal chance of enrolling in the program. Importantly, participants cautioned against using the term “lottery” to describe this process, given that their communities had a history of negative experiences with lottery-based programs, such as the local public housing selection process.

#### Other Support

Participants believed that the program should prioritize offering participants opt-in case management support. While they felt that engaging with case management should not be required with an unconditional program, they acknowledged that people have trouble accessing existing resources and navigating the benefits system.

### Design Sprint 2: Usability Testing

Eight participants from the first design sprint returned in December 2020 to test and provide feedback on all components of the ABP prototypes.

We elicited feedback on example recruitment documents. Participants preferred interest and referral forms that could be completed in 5 minutes or less. They suggested that the ABP website contain a portal to allow applicants to check their status from interest form submission to enrollment. To create a welcoming atmosphere, participants felt it was important for the website to have imagery representing Black and Pacific Islander people. This was especially important for the Pacific Islander participants because few programs tailored outreach specifically to this community. With this in mind, Expecting Justice collaborated with Black and Pacific Islander artists to design outreach materials such as flyers and social media posts. Expecting Justice consulted the San Francisco Pacific Islander Maternal Advisory Board about the best language to use to refer to the Pacific Islander population. They learned that the community felt most comfortable using “Pacific Islander,” “Pasifika,” or “Pasefika”—as opposed to “PI”—and consequently used these terms in their outreach materials. Participants also recommended that individuals be given a small welcome gift once selected. Participants felt that a list of local resources was insufficient in terms of offering support, and instead favored having program staff directly link participants to services (ie, a warm handoff).

During this design sprint, participants expressed concerns about people “misusing” the ABP funds. While we originally wanted to create the lowest documentation burden for applicants, participants in the second design sprint expressed a desire for stricter documentation requirements to ensure eligibility. These preferences mirrored those expressed in the needs assessment interviews and the first design sprint. In addition, participants discussed what would happen in the case of a pregnancy loss.

Participants also weighed in on their willingness to participate in research to evaluate the impact of ABP. They noted the factors that would make them more likely to participate in the research, including transparency in how the results would be used and knowing that the data collection would be conducted by Black and Pacific Islander community members.

### Final ABP Program Design

#### Overview

Through steps 1-4 of the formative community-centered research, we established the final program design of the ABP (Textbox 1). As a guaranteed income program, the income supplements were unconditional, which meant there were no requirements to receive or restrictions on the use of the funds. Recipients could use the funds in a manner that best supported their unique needs during pregnancy and postpartum.

**Final San Francisco Abundant Birth Project pilot elements, including the amount, duration, and disbursement of the income supplements, program eligibility criteria and assessment, participant selection, and additional support that resulted from the design process.**

**Program feature and selected design**

Amount and duration

- US $1000 per month for 12 months

Disbursement

- Direct debit card that could be used for cash or banking

Eligibility

- Identify as Black and/or Pacific Islander
- Living in San Francisco or recently displaced
- <27 weeks pregnant
- Annual household income of <US $100,000

Assessment of eligibility criteria

- Self-reported income
- Proof of residence
- Provider verification of pregnancy

Selection

- Random selection through the bimonthly abundance drawing
- Participants were re-entered into the drawing until they reached the third trimester

Additional support: abundance coaching

- Opt-in abundance coaching to support housing needs and connections to additional resources.

#### Eligibility and Verification

Eligibility criteria listed in Textbox 1 were assessed using a web-based interest form and confirmed in an intake interview that occurred over Zoom (Zoom Video Communications). Applicants were required to provide verification of pregnancy and documentation of residence in San Francisco to ensure the program served its prioritized population. The allowable documents to confirm residence included a California-issued ID, school documents, employer pay stubs, or utility bill statements. Gestational weeks of pregnancy were verified by a medical provider. The documentation requirements reflected the intention to strike a balance between honoring community voices and not creating overly burdensome eligibility requirements. Household income was self-reported and, to make eligibility assessment simpler for program staff, not adjusted by household size. The maximum income requirements were purposely selected to allow low- and middle-income families to participate, given the high cost of living in San Francisco and that many families may not qualify for other governmental benefits but struggle to meet basic needs. On the interest form, applicants were able to self-identify as Black and/or Pacific Islander, the communities most impacted by preterm birth in San Francisco.

#### Eligibility Changes Related to Pregnancy Outcomes

The design processes helped inform what would happen if a participant’s eligibility changed over the course of participation, particularly as it related to pregnancy outcomes. Using the expert opinion protocol, the ABP working group decided that in the event of a miscarriage, pregnancy termination, adoption, or death of the infant, ABP participants would continue to receive the income supplement for a 3-month grace period.

#### Implementing Randomized Selection

Bimonthly randomized drawings, called the “abundance drawing,” ensured that all eligible individuals had an equal chance of being selected for ABP within each drawing. If applicants were not selected into ABP, they remained in the eligibility pool until they were selected or entered into the third trimester of pregnancy and therefore became ineligible.

#### Additional Support: Abundance Coaching

ABP offered optional case management services called “abundance coaching.” In the needs assessment interviews, participants expressed a desire for more Black and Pacific Islander representation among staff from the local organizations that provided them with support. They also expressed previous stigmatizing experiences with government benefits programs. These perspectives informed the decision that all abundance coaches identify as Black and/or Pacific Islander to ensure racial and cultural concordance. Once enrolled, participants accessed optional support from abundance coaches who provided resource linkage and support with financial and other life goals. This included referrals to culturally specific community resources and providers. Unlike traditional case management which is often deficit-focused and paternalistic by directing and managing goals for participants, the abundance coaches provided individualized care and support based on the unique goals ABP participants set for themselves.

#### Evaluation

Using foundational strategies of equitable evaluation [49], we developed evaluation principles to abide by (1) leading with justice, (2) recognizing harm, (3) respecting autonomy, and (4) honoring community voice. We developed these principles based on ongoing work with the CRs and the responses from the interview and design sprint participants. The CRs, Expecting Justice collaborators, and research participants reported that too often Black and Pacific Islander people are excluded from decision-making in research and that there is a lack of transparency surrounding how data are used. The evaluation was opt-in for all ABP participants, based on the belief that the supplement should be truly unconditional, including research participation. The formative research also informed the decision that only CRs and other research team members who identified as Black and/or Pacific Islander would conduct surveys and interviews with participants.

## Discussion

### Principal Findings

Through a community-centered design process, we designed a novel guaranteed income program to center the needs of pregnant Black and Pacific Islander women and people in San Francisco. To our knowledge, no previous studies have qualitatively explored the financial, social services, and pregnancy and parenting experiences of these communities in San Francisco. Our needs assessment interviews revealed significant financial and life stressors experienced by Black and Pacific Islander people during and after pregnancy. Housing insecurity and previous poor experiences with social service programs were underscored by multiple participants. These findings are consistent with a report documenting that increases in San Francisco housing prices led to new concentrations of poverty especially for Black families from 2000 to 2015 [50]. Our findings also align with previous studies that report disproportionate unaffordable housing costs among people of color [51,52]. Additionally, the high cost of living in San Francisco left participants far above the federal poverty line ineligible for social services yet struggling to make ends meet. While all participants were enthusiastic about the potential of a guaranteed income program, they expressed concerns surrounding the eligibility and additional support needed to navigate the benefits system.

These experiences served as the foundation of the ABP prototypes and final program design. This formative process allowed for the establishment of eligibility and verification, supplement amount, disbursement options, and optional case management supports that were directly aligned with community members’ expertise and preferences.

### Strengths and Limitations

There are several strengths to this community-centered model of formative research and program development. Guaranteed income is a novel public health intervention, and the ABP program contributed to a swell of guaranteed income pilots in California and across the country [53-56]. Integrating community voice and expertise throughout the design process tailored ABP to the needs of the pregnant women and people it aimed to serve. We note that formative research with the CR model and shared decision-making requires more time and resources in the design phase, and institutions and academic hierarchies often do not allow space for this community-engaged work. Nonetheless, we strongly believe that community-centered formative research and program design ensures authentic engagement and leads to more relevant, responsive, and just programs and interventions. Our process can provide a roadmap for other programs to guide community co-design of an intervention.

Our results should also be considered in light of some limitations. Due to the COVID-19 pandemic, the needs assessment interviews and design sprints were implemented through videoconferencing only, and this mode of data collection may have affected the way participants engaged in the sessions. Participants’ experiences of pregnancy and parenting were also likely changed by the pandemic. Additionally, eligibility for the interviews and design sprints included the ability to speak and read English, so these findings may not capture the experiences of non–English-speaking Black and Pacific Islander pregnant people in San Francisco, particularly immigrants.

### Conclusions

The model of formative research to design ABP may be useful to other programs centering on community-driven, equity-oriented approaches. Interventions intended to address health equity should include experts with lived experience in the design process. Too often, well-intentioned programs do not meet the needs of recipients or center equity because of a lack of community participation in all elements of design.

The inclusion of CRs as integral members of the ABP staff was key to maximizing community involvement and shared decision-making. Ongoing staff and ABP working group meetings had designated spaces for CRs and community experts to advise ABP program implementation and evaluation design. Using the expert opinion protocol allowed for any ongoing issue to be decided with a consensus that prioritizes lived experience in developing solutions.

ABP aims to directly address the historical disinvestment of Black and Pacific Islander communities in San Francisco and positively impact birth outcomes in these communities through the provision of an unconditional income supplement. The COVID-19 pandemic has disproportionately impacted Black families, further underscoring the vital need for interventions that address structural deficiencies [57]. ABP applied a community-centered approach to design an intervention focused on the communities most impacted by maternal and birth disparities to reduce the effects of the racial wealth gap and economic insecurity among Black women. This novel approach and design process has great promise to reduce inequities in birth outcomes and center health and racial justice in antipoverty programs.

## Abbreviations

ABP: Abundant Birth Project
CR: community researcher
RAP: Rapid Assessment Process

## Data Availability

Data are not available due to their sensitive nature and the risk of deductive disclosure.
